# The Evolutionary Arms Race between Virus and NK Cells: Diversity Enables Population-Level Virus Control

**DOI:** 10.3390/v11100959

**Published:** 2019-10-17

**Authors:** Sarah K. A. Savoy, Jeanette E. Boudreau

**Affiliations:** 1 Department of Microbiology and Immunology, Dalhousie University, Halifax, NS B3H 4R5, Canada; sarah.ka.savoy@dal.ca; 2 Department of Pathology, Dalhousie University, Halifax, NS B3H 4R5, Canada

**Keywords:** natural killer cells, immunogenetics, KIR, killer immunoglobulin-like receptors, human leukocyte antigens, HLA, virus infection, education, licensing, cytomegalovirus, HIV, hepatitis C

## Abstract

Viruses and natural killer (NK) cells have a long co-evolutionary history, evidenced by patterns of specific NK gene frequencies in those susceptible or resistant to infections. The killer immunoglobulin-like receptors (KIR) and their human leukocyte antigen (HLA) ligands together form the most polymorphic receptor-ligand partnership in the human genome and govern the process of NK cell education. The *KIR* and *HLA* genes segregate independently, thus creating an array of reactive potentials within and between the NK cell repertoires of individuals. In this review, we discuss the interplay between NK cell education and adaptation with virus infection, with a special focus on three viruses for which the NK cell response is often studied: human immunodeficiency virus (HIV), hepatitis C virus (HCV) and human cytomegalovirus (HCMV). Through this lens, we highlight the complex co-evolution of viruses and NK cells, and their impact on viral control.

## 1. Introduction

Persons lacking natural killer (NK) cells or with defects in NK cell function present with recurrent viral infections, highlighting the crucial role of this population in virus control [1]. The hallmark function of NK cells is their ability to sense and eliminate targets based on a lack of “self” human leukocyte antigen (HLA) class I proteins (hereafter referred to as HLA). Although this “missing self” responsiveness initially defined NK cells, it is not the purview of all NK cells nor is it a binary function. The ability of an NK cell to mount an immune response against a “missing self” target is titrated on the avidity of interactions between inhibitory receptors and homeostatic concentrations of “self” HLA in a process called education, licensing, disarming or tuning [2,3,4].

Educated NK cells respond to infected, stressed or damaged cells that do not simultaneously provide an overriding signal for inhibition (i.e., that do not express HLA). NK cells with lower missing self reactivity are disarmed or uneducated; these cells are maintained in the NK cell repertoire but require a higher density of activating signals from the target cell or the local microenvironment to become activated. Since uneducated/disarmed NK cells are relatively insensitive to inhibition by “self” HLA, they are important effectors against infected cells that persist in HLA expression [2]. Adaptation of NK cells beyond education enables epigenetic alterations that establish “memory” NK cells that rapidly respond as powerful mediators to control recurrent infection [5,6].

In this review, we discuss the interplay between NK cell education and viral infection, highlighting the mechanisms of viral escape from and recognition by NK cells. We focus on the impact of NK cell education and highlight some of the strategies used by both NK cells and viruses in the evolutionary arms race to establish collaboration, host defense and viral persistence.

### NK Cell Education: Impacts of Diverse Immunogenetics

The ability of NK cells to kill tumour cells without prior sensitization was initially described in mice, and quickly recognized thereafter in humans [7]. This is remarkable: major histocompatibility complex molecules (MHC) in mice and humans (i.e., HLA) are extremely diverse, and the genes for the Ly49 (mouse) and killer immunoglobulin-like receptor (KIR) receptors that bind them derive from different founder genes [8,9]. In an outstanding example of convergent evolution, both fulfill the same purpose: modular recognition of “self” class I molecules [10].

*KIR* and *HLA* form the most diverse receptor-ligand partnership in the human genome, with each encoded on a highly polymorphic, polygenic locus [11,12,13]. KIR contain long (L) or short (S) cytoplasmic tails which generate inhibitory or activating signals, respectively. NK cell education can be influenced by both inhibitory and activating receptors and their interactions with subsets of HLA molecules. In general, inhibitory interactions act to lower the threshold for missing self reactivity, while activating partnerships can diminish NK cell reactive potential [2], ostensibly to avoid autoimmunity or impose stringent requirements for NK cell activation through these receptors.

The *KIR* gene locus is broadly classified as KIR-A or KIR-B based on the activating KIR gene content. This locus is comprised of a minimum of six KIR genes with *KIR2DS4* encoding the only activating receptor on KIR-A haplotypes. KIR gene loci can contain up to 14 KIR genes, including the activating genes that distinguish the KIR-B haplotypes [13,14,15]. These *KIR* genes segregate independently from those encoding *HLA*; thus, NK cell education differs even among related and closely-living individuals. Diversity in *KIR* and *HLA* exists throughout global populations, underscoring an evolutionary advantage that establishes responsiveness against an array of infected cell phenotypes [16,17,18]. While this may render individuals more or less competent for detecting particular phenotypes established by viral infections, it provides protection at the population level by introducing diversity difficult for a virus to subvert.

**Figure 1:**
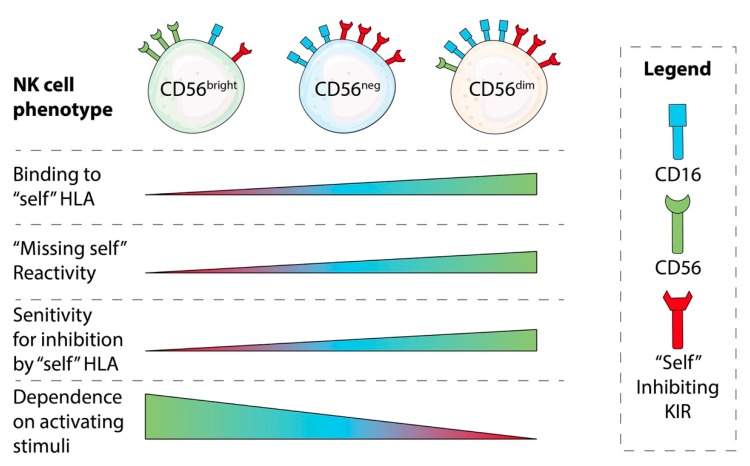
NK cell phenotypic variation and its impacts on NK cell education and interactions with viruses. NK cells are educated based on the interaction between their receptors and “self” HLA class I molecules. KIR, a family of receptors on NK cells that can be categorized as inhibitory or activating, bind to “self” HLA molecules. Highly educated NK cells are those whose inhibitory KIR strongly engage “self” HLA. This permits strong missing self reactivity, but also renders educated cells sensitive to inhibition against targets where HLA expression persists. By contrast, poorly or uneducated NK cells require strong activating signals to become reactive, but remain refractory to inhibition by HLA molecules. Hence, NK cell education creates a spectrum of diversity in NK cell effector responses. Some chronic viral infections, such as HCV, can skew an individual’s NK cell repertoire toward one comprised of cells with the phenotypic and functional characteristics of a naive population, including poorer cytotoxicity. During chronic infection, CD56bright NK cell populations diminish while CD56dim and CD56neg NK cell populations expand. The resulting NK cell populations display more CD16 receptors (which are responsible for ADCC), and increased activating receptor density, but are inefficient for eliminating infection.

In contrast to the exquisite recognition of “self” HLA molecules by T cells, NK cells recognize conserved epitopes on groups of HLA molecules. The major KIR and HLA partnerships that interact to educate NK cells are shown in Table 1. The avidity of inhibitory interactions at steady state calibrates their threshold for reactivity [19,20,21] (Figure 1). NK education therefore exists on a continuum that can be measured as responsiveness against an HLA-negative target cell from relatively non-responsive (and refractory to inhibition), to highly responsive (and highly sensitive to inhibition). Conserved receptors for non-HLA ligands (i.e., TIGIT) [22], and those that bind HLA and its components (i.e., NKG2A, LILRB1) [23] each contribute to NK cell education alongside the polymorphic receptors (i.e., KIR). Finally, NK cell receptors are expressed and co-expressed codominantly within the NK cell repertoire, creating an array of NK cells with differing patterns of education and reactive potential.

**Figure 2:**
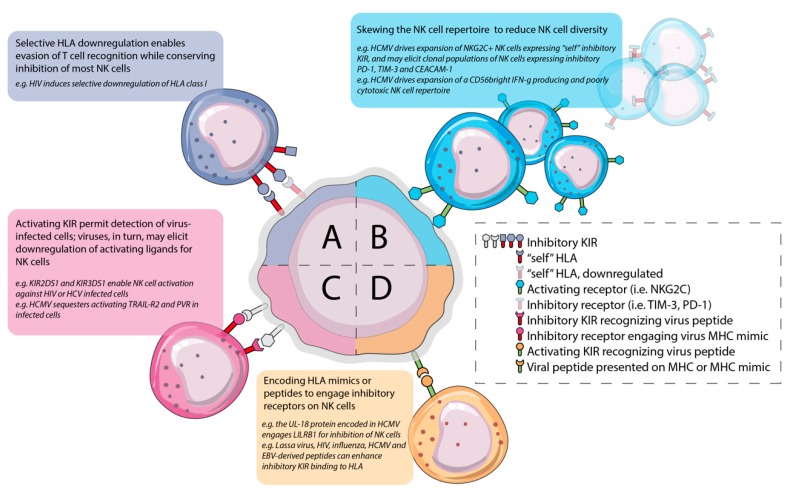
Interplay between virus infection and NK cells. Viruses and NK cells have co-evolved to enable virus persistence, host defense and symbiosis. Four examples are shown in this figure where the center cell represents an infected cell. **A.** Some viruses can selectively downregulate HLA molecules to avoid recognition by T cells. While this may create a target for the educated subset of NK cells, uneducated NK cells will not productively detect downregulation of HLA. Hence, the virus is taking a calculated risk that any given host may not be able to detect the loss of HLA that it induces. **B.** Viruses can skew the NK cell repertoire, reducing its diversity. In some instances, viruses (i.e., HCV) drive expansion of a CD56-negative exhausted NK cell population; others (i.e., HCMV) induce expansion of an NKG2C+ “adaptive” NK cell population that may better equip individuals for responsiveness to subsequent infections. **C.** Viruses (i.e., HCMV) may encode HLA mimics or peptides that strengthen binding to inhibitory receptors to decrease the NK cell response to virus infection. **D.** Activating KIR, which typically bind poorly to “self’ HLA may permit NK cell recognition of cells presenting virus-derived peptides.

Depending on the virus and host HLA, infected target cells may persist in HLA expression, induce its downregulation, or modify HLA with KIR-antagonizing peptides [24]. Hence, diversity in education and HLA binding sensitivity could have important impacts on the ability of NK cells to remove virally-infected targets. No compound *KIR-HLA* genotype is universally beneficial or detrimental for virus control. The relative susceptibility of certain genetic combinations to particular virus infections is likely the cost of a constant co-evolution between NK cells and viral infection (Figure 2).

## 2. Taking a Calculated Risk: Genetic Variation between Hosts Creates an Opportunity for a Variety of Infection Outcomes

The adaptive immune system detects virus infection through presentation of virus-associated antigens to T cells on HLA molecules. To avoid recognition, some viruses induce downregulation of HLA, but this creates a target for educated NK cells, so many viruses have evolved to selectively or incompletely eliminate HLA, which preserves inhibition of NK cells. In this way, virally-infected cells evade recognition by T cells and experience efficient or inefficient NK cell responses that differ between hosts, which permits viral persistence and spread throughout populations.

The susceptibility or resistance of an individual to infection by a given virus is closely related to the capability of their NK cells to sense and respond to virus-induced changes. Since NK cell function differs with education and compound *HLA-KIR* genotypes, individuals will be differently able to combat infection. In those carrying NK cell subsets capable of detecting the changes introduced by a given virus, the virus will be eliminated. In others who lack NK cells appropriately educated to control them, the virus may instead establish chronicity.

### 2.1. HIV Induces HLA-Bw4 Downregulation, Creating a Target for Educated KIR3DL1+ NK Cells

The human immunodeficiency virus (HIV) nef protein binds the cytoplasmic region of HLA-A and -B molecules, causing a decrease in their expression at the cell surface [25,26,27,28]. HLA-A is the predominant HLA that engages T cells and the primary target of nef, so its downregulation enables infected cells to escape T cell-mediated recognition [27]. Downregulation of HLA-B molecules—generally stronger agents of NK cell education and inhibition than HLA-A—selectively creates a target for the subset of NK cells educated by KIR3DL1 [19,26]. In the absence of this inhibition, ligands for the activating NK cell receptors DNAM-1 and PVR that are induced by the stress caused by virus infection rebalance NK cell reactivity toward clearance of HIV-infected cells [29].

HLA-B molecules carrying the Bw4 motif interact with KIR3DL1 (hereafter referred to as HLA-Bw4). HLA-Bw4 molecules can be subdivided based on the amino acid at position 80—isoleucine (80I) or threonine (80T), with correlations to high and low cell surface expression and consequences on binding avidity with different KIR3DL1 isoforms [19,30]. Likewise, the KIR3DL1 alleles exist as three functional subtypes expressed at null, low and high cell surface densities, with impacts on NK cell education [19,31,32]. Partnerships that bind with high avidity (i.e., KIR3DL1-high isoforms with Bw4-80I) exhibit stronger missing self reactivity, but also a higher sensitivity to inhibition compared with the other pairs.

In HIV-infected individuals, an important role of highly educated NK cells is supported by genetic studies: those encoding the genes for the strongly-educating combination of *KIR3DL1-high* and *HLA-Bw4-80I* molecules progress to acquired immune deficiency syndrome (AIDS) slower than those who lack NK cells strongly educated by HLA-Bw4. In contrast, patients with combinations of genes that predict for poorly-educated or uneducated KIR3DL1^+^ NK cells (i.e., *HLA-Bw4*-negative), progress rapidly to AIDS [33]. This reactivity corresponds with the magnitude of missing self reactivity endowed to NK cells through education: the NK cells that exhibit the highest missing self reactivity are also the most potent responders to HIV-infected autologous CD4^+^ T cells [19]. Taken together, these observations demonstrate how NK cells, differentially educated in each host, vary the outcome of HIV infection through an education process that calibrates sensitivity to loss of HLA expression.

### 2.2. Downregulation of HLA-C by HIV Creates a Target for NK Cells Educated by KIR2DL1/2/3

In addition to HLA-A and HLA-B, HLA-C expression can be downregulated by HIV infection, a process facilitated by the Vpu protein [34,35]. Since HLA-C likely evolved primarily for interactions with NK cells (not T cells) and nearly every HLA-C molecule encodes a ligand for KIR, this may represent a strategy from HIV that permits evasion of NK cell reactivity [24,34,36,37]. In agreement with this, HIV strains elicit a range of capabilities for HLA-C downregulation, from no downregulation among the common laboratory strain, NL4-3, to up to three-fold reductions driven by primary HIV isolates, that correlates to decreased binding by KIR [34].

HLA-C is recognized by the inhibitory receptors, KIR2DL1, KIR2DL2 and KIR2DL3, which are comparatively less diverse than KIR3DL1. *KIR2DL3* is exclusively encoded on KIR-A haplotypes, and segregates as an allele with *KIR2DL2*, a KIR-B haplotype-defining gene [15]. Both KIR2DL2 and KIR2DL3 bind HLA-C1 isoforms and KIR2DL2 additionally binds a subset of HLA-C2 isoforms [38,39,40]. Thus, KIR-A and -B haplotypes have both evolved to enable recognition of both HLA-C1 and HLA-C2 isoforms [37]. Like HLA-Bw4, HLA-C is expressed at different levels between donors even without HIV infection [41]. The dose of HLA-C2 ligands in the genome correlates positively with the strength of education among KIR2DL1+ NK cells [42]. Although the strength of binding differs between KIR2DL2/3 and their ligands, impacts on NK cell education are unclear.

Loss of HLA-C expression does drive NK cell reactivity toward autologous HIV-infected CD4+ T cells, especially among the educated KIR2DL populations, where loss of HLA-C triggers a missing self response [34]. The magnitude of responsiveness between donors is variable, consistent with a previous finding that the density of HLA-C on the cell surface is correlated with protection against HIV [41]. Together, these results support the conclusion that NK cell education, by calibrating the sensitivity with which NK cells sense loss of HLA, permits a spectrum of missing self reactivity relevant to detecting and eliminating HIV-infected cells. Thus, for HIV, outcomes of infection differ between people, from control to latency or fulminant infection, based on the education of NK cells. At the population level, this is a clever way to simultaneously facilitate viral persistence and transmission within a population.

### 2.3. Immunogenetic Diversity Predicts Susceptibility, Resistance and Disease Processes in Hepatitis C Virus Infection

Hepatitis C (HCV) infection results in an array of outcomes: some, including people who use drugs (PWUD) are chronically exposed but remain seronegative; others clear an acute infection and still others harbour a chronic infection. Like for HIV, differences in viral control have been associated with variable NK cell education.

Genetic association studies reveal enrichment of persons with compound *KIR-AA HLA-C1/C1* haplotypes among those resistant to developing chronic HCV infection and pathology, including exposed seronegative individuals [43,44,45]. This same genetic combination has been associated with continuous response to anti-HCV therapy using interferon alpha and the antiviral, ribavirin [46,47]. Among PWUD, both homozygosity for high-expression level *HLA-C1* molecules [48], and the combination of *KIR3DL1* and *HLA-Bw4-80T* are associated with resistance to chronic HCV infections [49].

Unlike HIV, where the most strongly educated NK cell populations associate with antiviral activity, the partnerships of KIR3DL1 and HLA-Bw4-80T or HLA-C1 with KIR2DL3 exhibit only moderate binding affinities and relatively weak education [19,39]. Further research is needed to understand the mechanistic interactions between NK cells and HCV-infected targets, including how HCV infection influences HLA expression. One interpretation of the genetic epistatic interactions may be that HLA expression persists after HCV infection and the resulting poor binding between KIR and HLA conveys relatively weak inhibition of NK cells, allowing NK cell activation to proceed. Further study will be required to understand the dynamic nature of HLA expression following HCV infection and the consequences on recognition by differently-educated NK cells.

### 2.4. The KIR Strike Back: Activating KIR Are Associated with Virus Control

Although NK cell education was initially defined based on missing self reactivity and interactions between inhibitory receptors and HLA, the highly-homologous activating KIR are now known to impact the education of NK cells. In particular, activating receptors can lower the NK cell threshold for reactivity. KIR2DS1 is activated by HLA-C2, but KIR2DS1+ NK cells are rendered anergic in HLA-C2 homozygous individuals [50,51]. This relative anergy can be overcome by strongly-educating inhibitory KIR2DL1-HLA-C2 partnerships [42].

Other activating KIR are now known to bind with HLA isoforms under specific conditions. For example, KIR3DS1 interacts with HLA-F in its open conformation, with some dependency on peptide sequences [52,53,54], and KIR2DS2 can bind HLA-A*11 and subsets of HLA-C molecules in a peptide-specific manner [24]. Whether interactions between HLA and these other activating KIR can also promote tolerance among NK cells is not yet understood.

Activating KIR typically bind their ligands with lower affinities than their inhibitory KIR counterparts, but their interactions may become highly relevant in the context of viral infection. KIR-B content, including KIR2DS1 and KIR3DS1 in particular, is associated with resistance to HIV infection and HCV chronicity and development of hepatocellular carcinoma [43,55,56,57]. In HIV elite controllers (i.e., those infected with persistently low viral loads), compound genotypes of *HLA-C2* and activating *KIR2DS* genes (i.e., KIR-B content) are enriched, as is the presence of high-density isoforms of HLA-C. This together suggests a benefit of activating KIR in the presence of their ligands [41,57].

Intriguingly, HLA-F, in its “closed” confirmation acts as an inhibitory ligand (via the LILRB1 receptor) [58]. During virus infection, however, HLA-F is upregulated on HCV-infected hepatocytes or HIV-infected CD4+ T cells, and provides an activating signal that triggers cytokine production and cytotoxicity of KIR3DS1+ NK cells [53,59]. Interactions between KIR3DS1+ NK cells with infected targets promote IFN-γ production and degranulation [59,60]. Hence, the activating KIR3DS1 receptor may provide an advantage to support inflammation and consequently, continued viral control. Thus, like HLA-C, HLA-F acts in a context-specific and highly dynamic manner. Not all haplotypes or individuals encode the activating *KIR*; in those who do, they may provide a distinct advantage to combat viral infection.

The understanding of activating KIR is underdeveloped compared with that of the inhibitory KIR, but epistatic interactions and the conservation of KIR-A and -B haplotype diversity suggest a distinct evolutionary advantage for both of these sets of receptors in combatting viral infection. Indeed, both the activating and inhibitory KIR, together with their HLA ligands exhibit substantial diversity that impacts control and persistence of viral infections.

## 3. Distract and Redirect: Infection Leaves Stable Imprints on the NK Cell Repertoire that Impact Viral Control and Enable Chronic Infection

Extensive variability within and between individuals is established by variegated expression of activating and inhibitory receptors [61,62]. This provides resilience against an array of challenges. In response to virus infection, certain NK cell populations may expand, at the cost of this diversity. This expansion, attempting to clear or adapt to a particular infection, may lead to NK cell exhaustion and/or a loss of resilience against subsequent challenges.

### 3.1. HCMV Infection Drives Expansion of Adaptive/Memory Self-KIR+ NKG2C+ NK Populations and May Impact the Outcome of Co-Infection

More than any other virus, human cytomegalovirus (HCMV) has been scrutinized for its ability to induce population-wide alterations in the NK cell repertoire. Persons chronically infected with HCMV exhibit expansion of an epigenetically-differentiated genome (similar to that of an effector T cell) that corresponds with diminished expression of the transcription factor, promyelocytic leukemia zinc finger (PLZF), the transmembrane adaptor protein FcεRIγ, the tyrosine kinase SYK, and the intracellular adapter EAT-2 [5]. Phenotypically, these “adaptive” or “memory” NK cells exhibit enhanced expression of NKG2C (with concomitant loss of NKG2A), CD2, LILRB1, LFA-1 and self-specific KIR [5,63]. Compared with canonical NK cells, these adaptive NK cells display enhanced capabilities for antibody-dependent cell-mediated cytotoxicity (ADCC), but are poorer at natural cytotoxicity functions and IFN-γ production in response to pro-inflammatory cytokines [5].

NK cell memory was first defined in mice infected with murine cytomegalovirus (MCMV), where a viral MHC class I mimic, m157, is detected by the mouse Ly49H activating receptor. This interaction is essential for viral control by NK cells [64,65,66]. The interaction between Ly49H and m157 enables potent NK cell reactivity against MCMV, but skews the NK cell repertoire toward an adapted one and diminishes activation of anti-MCMV CD8^+^ T cells [67,68]. Hence, in mice, incomplete control of MCMV establishes a long-term interaction with host NK cells that is associated with memory/adaptation among the NK cell population.

In humans, chronic HCMV infection also leaves stable imprints on the NK cell repertoire [69], principally through expansion of NKG2C+ NK cells that co-express self-specific inhibitory KIR, the inhibitory LILRB1 receptor and exhibit decreased expression of the activating receptor NKp30 [42,63,70]. NKG2C copy number corresponds with susceptibility to HCMV reactivation following hematopoietic cell transplantation, suggesting that NKG2C may influence initial control of HCMV upon reactivation [71]. However, unlike Ly49H in mice, NKG2C is dispensable for the generation of adaptive NK cells in response to HCMV infection: individuals carrying a homozygous deletion of NKG2C that prevents protein expression still mount adaptive NK cell responses that exhibit a phenotypic, functional and epigenetic profile of adaptive NK cells [72,73,74]. NKG2C-null adaptive NK cells exhibit higher CD2 expression than their NKG2C-sufficient counterparts, suggesting that CD2 and NKG2C may have overlapping roles in the generation of adaptive NK cell responses [73].

The relationship between HCMV and NK cells may be symbiotic. In patients undergoing hematopoietic cell transplantation for treatment of leukemia, reactivation of HCMV drives rapid development of adaptive NK cells, which is associated with better protection against leukemic relapse [75,76]. Pre-infection with HCMV (and the resultant population of adaptive NK cells) may facilitate stronger anti-viral NK responses, marked by faster, stronger proliferation of adaptive NK cells upon infection with HIV [73,77]. Although the mechanism through which HCMV pre-infection fosters improved control of heterologous and subsequent infection remains to be conclusively demonstrated, a potential benefit may be the improved ADCC mediated by adaptive NK cells as they trade the FcεRIγ signal adaptor for the more efficient CD3ζ [78]. Pre-infection with HCMV drives expansion of a subset of NK cells with zero or low expression of FcεRIγ in some individuals upon subsequent infection with HCV. This phenotype is associated with diminished pathology, decreased liver damage and decreased fibrosis compared with those that are HCMV-negative at the time of HCV infection [79,80,81]. Thus, HCMV infection, and the related adaptation of NK cells can permit better innate control of viral infections, possibly by preparing a population of NK cells that are highly efficient at ADCC.

### 3.2. Chronic HCMV Infection is Associated with Dysfunction and Exhaustion in NK Cells

Adaptive NK cells that result from chronic HCMV infection may compromise immune function by promoting NK cell exhaustion. Recently, NK cell adaptation and HCMV infection have been shown to enhance NK cell expression of PD-1, TIM-3 and CEACAM-1 (CD66a), inhibitory molecules whose signaling slows NK cell proliferation and diminish NK cell pro-inflammatory activities [82,83]. In HIV, a well-documented immune exhaustion occurs, but this may represent an exacerbation from HCMV, with which the vast majority of HIV+ individuals are co-infected [84].

Like HCMV, chronic infection with HCV leads to stable imprints on an individual’s repertoire that reduce its overall diversity and change the function of the NK cell population (Figure 1). In HCV, these changes favour chronic maintenance of viral load. In healthy individuals, the CD56^dim^ population dominates over CD56^bright^, but during HCV infection, this reverses with an increase in CD56^bright^ NK cell numbers, and with increased expression of CD69, CD95 (FasL), NKp30 and NKp46 [85,86,87]. The CD56^bright^ population exhibits greater IFN-γ production but poorer cytotoxicity. Thus, the expansion of this population represents naïve NK cells with increased activating receptor density that are inefficient for combatting infection [85].

The CD56^dim^ population of NK cells is important in clearance of HCV-infected cells. They are typically found at high levels in highly exposed HCV seronegative PWUD [88]. In contrast, HCV infection cannot always be eliminated by NK cells; a shift in the NK cell repertoire has been observed within individuals who develop chronic HCV infection. Several studies have identified an increase of CD56^negative^ and CD16^+^ NK cells in chronically infected individuals, which exhibit decreased cytotoxicity and low perforin expression leading to weakened target cell killing and persistent infection [89,90,91]. Although HCV-infected persons display a normal array of inhibitory KIR that would be associated with NK cell education and missing self responsiveness, their NK cells exhibit reduced functionality, leading to unsuccessful elimination of HCV infected cells [89,92]. The mechanisms of this failure in NK cell responsiveness remain to be studied, but this observation suggests adoption of an exhausted NK cell population in response to chronic HCV infection.

## 4. Resetting the Balance to Favor Inhibition over Activation

Virus infection can hinder NK activation by changing the landscape or ligandome of the target cell. In some instances, viruses encode HLA mimics or force expression of proteins that bind KIR or other inhibitory receptors to signal NK inhibition. Other viruses alter the interactions between KIR and HLA by presenting a peptide that changes their steric interactions. By skewing the immune response toward inhibition or away from activation, viruses can persist in the presence of NK cells.

### 4.1. Chronic Infection Induces Inhibitory Receptor Expression and NK Cell Exhaustion or Diminishes Activating Receptor Expression

In HCV, a variety of inhibitory pathways are induced during acute and chronic infection. In addition to reduced total numbers of NK cells in the circulation, patients with chronic HCV exhibit higher levels of PD-1 [93]. The HCV core protein stimulates NK cells to increase release of IFN-γ, which in turn results in increased PD-1 expression that is associated with diminished NK cell responsiveness, and higher viral loads [93,94]. Levels of other inhibitory factors, including CEACAM-1/CD66a and CD81 are similarly elevated in persons chronically infected with HCV [95,96,97]. These molecules, in turn, support viral persistence and progression of infection to hepatocellular carcinoma [95,98]. Together, these alterations reflect a general and non-specific skewing of the NK cell response toward inhibition that permits chronic infection.

HCMV encodes an array of inhibitory molecules for NK cells; among them is the UL-18 protein, an HLA mimic that binds the LILRB1 inhibitory receptor on NK cells. The interaction between UL-18 and LILRB1 steers the NK cell away from killing, which allows HCMV to evade NK cell recognition and sustain chronic infection [99]. Between individuals, polymorphisms in the *LILRB1* gene titrate the strength of binding to UL-18, with impacts on the strength of inhibitory signals generated [100]. In HCMV-naïve patients receiving kidney transplants from HCMV+ donors, variations in LILRB1 that permit greater NK cell inhibition are associated with trends toward lower HCMV viremia and reactivation [100]. Collectively, the inhibitory input triggered by UL-18 + LILRB1 interaction and the varying strength of inhibition governed by *LILRB1* polymorphisms, promote successful evasion from NK cell killing to establish chronic HCMV infection.

In addition to enhancing inhibition, HCMV can also diminish availability of activating signals to promote immune evasion. The HCMV glycoprotein UL141 sequesters the target cell death receptor, TRAIL-R2, and the nectins PVR (CD155) and CD112 and tags the latter using the protein US2 for degradation by the proteasome [101,102,103]. Loss of PVR and CD112 on the surface of the target cell has a dual impact on NK cell function: NK cell adhesion and migration are impeded, resulting in NK cells unable to interact with the infected cell [103]. Moreover, PVR and CD112 function as ligands for the NK cell activating receptor DNAM-1 [102]. Hence, the downregulation of nectins as well as TRAIL-R2, creates a deficit in NK cell activating signals that permits persistence of HCMV.

### 4.2. Virally-Encoded Peptides Alter KIR-HLA Interactions to Favour Inhibition

Additional changes in KIR-HLA interaction can occur when virally-encoded peptides are loaded onto HLA. Unlike the T cell receptors, which are exquisitely sensitive to specific peptide sequences presented in “self” HLA, KIR bind outside of the peptide binding groove and are not known to be peptide-specific. Nevertheless, KIR binding is impacted by peptide sequences, which can alter the HLA conformation, with impacts on the strength of interactions between KIR and HLA. In turn, these changes can strengthen inhibitory interactions, thwart activating signals or permit progression of antiviral activity; all of this may be influenced by a person’s compound *KIR-HLA* genotype.

At steady state, the KIR3DL2 receptor is not known to bind with any HLA. Several peptides derived from HIV, influenza, HCMV or Epstein-Barr virus loaded into HLA tetramers (but not peptides eluted from uninfected cells) can bind with KIR3DL2 [104], suggesting that viral peptides may modify KIR-HLA interactions to favor inhibitory signaling in NK cells. Likewise, tetramers loaded with peptides derived from HIV or HCV stabilized binding of HLA-A*24 to KIR3DL1 isoforms [105]. Similarly, the HCV core peptide can bind to HLA-C to increase KIR2DL1 receptor affinity for its HLA-C ligand, resulting in increased inhibitory signaling [106]. Lassa virus peptides similarly bind with HLA-C ligands and enhance binding to KIR2DL1 and KIR2DL2 [107]. Persons succumbing to Lassa virus infection are enriched for the educating partnership of *KIR2DL2* and *HLA-C1* compared with healthy controls and infected survivors, which suggests that inhibitory peptides encoded by Lassa and presented on HLA-C1 can indeed inhibit or slow antiviral NK cell responses [107]. Taken together, these examples highlight that virally-encoded peptides that bind HLA and support engagement of inhibitory receptors may favor viral persistence.

Peptide variations do not always prevent inhibition, and their impacts can vary based on the education of a person’s *KIR-HLA* genotype. For example, the immunodominant peptide from the HIV gag protein evades recognition by T cells through mutation, but stabilizes binding to the HLA-Bw4 isoform, HLA-B*57. In individuals co-expressing KIR3DL1, this altered peptide prevents binding (and therefore inhibitory signaling) [108]. Peptide binding in HLA molecules can also serve to permit HLA binding to activating KIR. For example, a peptide derived from the HCV helicase can be presented in HLA-C*01 and detected by KIR2DS2+ NK cells, triggering activation [109]. The same genetic combination similarly enables recognition of a conserved helicase peptide sequence in flaviviruses [109]. Hence, although viruses may tailor peptides to support NK cell inhibition over activation, in those individuals activating KIR, this strategy could backfire as NK cells recognize viral peptide-loaded HLA molecules as activating ligands.

## 5. Summary and Future Directions

NK cells’ ongoing interactions with viruses are complex and foster their co-evolution; imprints of this co-evolution are visible as viral adaptations and diversity in NK cell immunogenetics. Variation in HLA diversifies the range of peptides that can be bound and presented both to T cells and NK cells. These alterations in HLA also impact the strength with which KIR molecules are bound, and the avidity of these overall interactions by impacting the density of their cell surface expression. Viruses, in turn, may alter NK cell recognition by forcing inhibition, encoding inhibitory mimic molecules or playing the odds to simultaneously avoid recognition by T cells and NK cells through selective downregulation of particular HLA molecules that are not present in all individuals in a population. Increasing resolution and techniques to assess NK cell immunogenetics, function and viral complexities is shedding light on NK-virus interactions.

The relationships between viral infection/co-infection with diverse NK cell function driven by NK cell education remain to be extensively studied. Given the impact of NK cell education on the potential outcomes, it will be important to study these interactions in human populations. Populations who are at-risk for viral infection through repeated exposure, immunocompromise or risk behaviors are identifying how the differences in NK cell between individuals impact resistance or susceptibility to infection. Prospective studies in at-risk populations could lead to more precise approaches to vaccination, prevention and therapy that work with a person’s NK cell repertoire to facilitate durable virus control. In the case of HCV, the increasing use of direct acting antivirals as a cure affords an opportunity to understand how the NK cell repertoire changes in the presence or absence of virus infection. Likewise, HCMV-seronegative patients undergoing transplantation of HCMV+ donor cells and organs afford the opportunity to study virus-NK interactions in real time.

Differences among NK cell education, phenotype and function predict outcomes of virus infection and highlight opportunities for antiviral strategies that collaborate with NK cell reactive potential. These studies and strategies should be considered in light of patients’ compound *KIR* and *HLA* genotypes, now known to dramatically alter the NK response to, and outcomes of, virus infection. That NK cells can exhibit long-lived memory and virus-specific immune responses suggests that they may be mediators or targets for effective vaccination. In particular, strategies to elicit adaptive NK cell populations or promote NK cell activation by vaccines especially in the therapeutic or acute context are all the subjects of active investigation. Further study of NK cell signaling and the components of activation and regulation that influence virus infection and clearance might assist in pharmacologic manipulation of NK cells or adoptive cell therapy to maximize their antiviral contributions.

## Figures and Tables

**Table 1:** Educating KIR-HLA partnerships.

KIR	Ligand	Notes
Inhibitory partnerships		
KIR2DL1	HLA-C2	
KIR2DL2	HLA-C1 (major); HLA-C2 (minor)	HLA-C1 ligands carry Asp77; HLA-C2 ligands carry Lys77
KIR2DL3	HLA-C1	
KIR3DL1	HLA-Bw4	HLA-Bw4 epitopes can be further subdivided based on the amino acid at position 80 (Ile or Thr) with impacts on NK cell education
Activating partnerships		
KIR3DS1	HLA-F	KIR3DS1 is known to bind HLA-F, but its impact on education is unknown.
KIR2DS1	HLA-C2	Individuals homozygous for HLA-C2 exhibit tolerized KIR2DS1+ NK cells
KIR2DS2	HLA-A*11 (weak and peptide dependent)	
